# Context as politicised psycho‐geographies: The psychological relationship between individual, politics, and country

**DOI:** 10.1111/bjso.12848

**Published:** 2025-01-28

**Authors:** Geetha Reddy, Ilka H. Gleibs

**Affiliations:** ^1^ The Open University Milton Keynes UK; ^2^ University of Cape Town Rondebosch South Africa; ^3^ London School of Economics and Political Science London UK

**Keywords:** coloniality, context, identity, multiculturalism, race, space

## Abstract

This paper sheds light on how spaces become contested sites for identity construction and negotiation to take place. Applying the Social Representations Approach, a qualitative study of 10 focus group discussions (*n* = 39), was conducted in Singapore, Malaysia and the UK to explore how, and why racialised identity construction changed in each socio‐political context. The study challenged two underlying assumptions in social psychology: (1) that the meaning of the racialised category holds constant across time and space, and (2) there exists a pan‐racial identification among Asian identities, for example, which at times allows for racialised categories to be manipulated as variables. We argue that the distinction between the country that the racialised identity originates from, country of birth (or citizenship) for the individual and country that the individual manages the identity in, is important in understanding the changes in the psychology of racialised identities. By taking into consideration the interplay of temporality, space, social relations and social systems, this paper presents a contribution in the form of the concept “politicized psycho‐geographies”.

## INTRODUCTION

When individuals migrate to a new country, they are constructing, and re‐constructing their identities as they encounter different knowledge systems in new contexts and this psychological process warrants our attention because international migrant numbers continue to increase worldwide (UN Migration, 2024). Within social psychology, it is understood that how much one identifies with a specific (social) category changes as the context itself changes. Yet the change in identity content and meaning to the individual is under‐researched, and “context” itself is often loosely conceptualised. Furthermore, there is an underlying assumption that the meaning of a racialised category holds constant across time and space and across diverse groups (Goh & McCue, 2021; Iwamoto & Liu, 2010).

This paper aims to address these assumptions and loose conceptualisations by examining racialised identity construction among both majoritised and minoritised racialised groups from two countries, Malaysia and Singapore, living in three countries, namely Malaysia, Singapore and the UK. This paper forms part of a larger project understanding the strategic construction of racialised identities among Malaysians and Singaporeans (Reddy, 2017, 2019; Reddy & Gleibs, 2019) and focuses on understanding how, and why, racialised identity constructions change in different socio‐political contexts. As such, it contributes to our understanding of the interrelations between how geopolitical histories become embodied by individuals' identities and the spaces they inhabit, and how changes to the latter impact experiences, and expressions of, the former. We first examine key theoretical frameworks and empirical studies related to identification and identity content. Next, we look at racialised and national identities in context, focusing on how migration as a social psychological phenomenon draws these two constructs together. Then, we introduce our conceptualisation of geopolitical contexts to offer a broad overview of the research context before discussing the two studies that are presented in this paper.

## IDENTITY IN CONTEXT

This paper contributes to the existing research on racialised identity construction by examining racialised identification and racialised identity content change across different contexts using Social Representations Approach (SRA; Elcheroth et al., 2011). SRA enables an examination of process (how one identities), and content (what the identity means). With these two aspects in mind, we explore whether changes in one's socio‐political context, result in changes in one's construction of racialised identity (*change in identity content*) as well as changes in how much one identifies with their racialised identity (*change in levels of racialised identification*) at the same time.

### Identification

Research into identities, especially within the Social Identity Theory tradition (Tajfel & Turner, 1979), has focused on motivations behind identity categorisation, as well as how levels of identification change across contexts. Herein, a leading view of social identity is that it is both individual and social, is relational because we define ourselves based on comparisons with Others, and provides a basis for shared social action as we share identity with Others (Reicher et al., 2010). To this end, studies of racialised, religious, and national identities have examined shifts in cognitive processes (such as levels of identification among individuals) in relation to shifting contexts. For example, Khan et al. (2014) showed how a change in one's physical location resulted in an increase in religious identification. Similarly, different societies have been shown to promote different levels of racialised identification; a society that is more open to embracing multiculturalism leads to stronger racialised identification among minority group members, compared to a society that prefers assimilation, which leads to weaker racialised identification (Verkuyten, 2007). Thus, the strength of racialised identification changes as context changes. However, it is less clear if the nature of the identity (what it means), also shifts as a consequence of changing contexts.

### Identity content

Turner (1999) and Moloney and Walker (2007) have argued for the incorporation of the analysis of identity content into studies on identity processes as it has not been a key focus within the social identity tradition. People are active meaning‐makers and not responding passively to stimuli (Elcheroth et al., 2011). Therefore, identity content is important in understanding identity because the meanings (or content) associated with any social identity are a result of “our collective history and present” (Reicher et al., 2010; p. 45). Social psychological research focusing on collective action and intergroup relations has indeed focused on identity content as a means of, for example, differentiating the psychological crowd that shares an identity from the physical crowd (Reicher, 1984; Templeton et al., 2018) as well as how some forms of intergroup behaviour depend on the content of the identity (Livingstone & Haslam, 2008; Turner‐Zwinkels et al., 2017).

Contrarily, Social Representations Theory (SRT; Moscovici, 1984) has highlighted the importance of the content of identities. Social representations are a function of social identities, and the central core of a representation consists of “one or several elements that give the representations its meaning” (Abric, 2001; p.43), thus placing identity content at the heart of social psychological understanding of identity. In her research on African American identity, Perkins (2006) showed that while American participants from Africa, the Caribbean and people of African‐descent who were born in the US self‐identified as African American, the meanings associated with that categorical label had distinctly different profiles. For example, African and Afro‐Caribbeans downplayed the pervasiveness of discrimination in society. While racialised identification was the same, identity content differed across the racialised groups. This goes to show that examining racialised identity content is a meaningful exercise for the social psychological understanding of racialised identities. In fact, why and how changes in content occur are viewed as fundamental questions within the SRT paradigm (Moscovici, 2001). More recent work has used SRT to understand meaning‐making of racialised identities, migrant identities and citizenships showing us the interplay of representations, identity content and context (Kadianaki, 2014; Kadianaki & Andreouli, 2017; Lukate & Foster, 2023). To illustrate, representations of citizenship differ between citizens and migrants in Greece based on interpretations of legality and the perceived assimilation of migrants, showcasing how tensions related to identity content can lie within the same socio‐political context (Kadianaki & Andreouli, 2017).

We argue that a social psychological perspective of racialised identity construction encompasses the process of identity construction, the content of identity construction, as well as the motivations of identity construction, in the presence of Others, implied, imagined and present. Importantly, racialised identities are not only constructed by the individual, they are co‐constructed with Others. This multi‐faceted perspective of racialised identity construction looks at *what* racialised identity *does* for individuals and *how*, *when*, *where*, *why* and *with whom* these racialised identities are constructed. Thus, racialised identity construction is as much social cognition, as it is social construction. Howarth (2002b) has outlined how Social Identity Theory (SIT; Tajfel & Turner, 1979) and Social Representations Theory (SRT; Moscovici, 1984) can be used together to understand racialised identity construction among teenagers in Brixton, UK. In particular, she has shown how the presence of Others brings about awareness of racialised identities and discrepancies in the content attributed to these identities. Other psychologists have combined SIT and SRT in different ways in the study of racialised identity construction in Australia (Augoustinos & Riggs, 2007) and in the US (Philogène, 2007). We build on this tradition of using Social Representations Approach (SRA; Elcheroth et al., 2011) and extend it to study racialised identity construction among Malaysians and Singaporeans.

Because identification and identity content is rooted in context, some psychologists have argued that an individual's identity is motivated to adapt to a social context, specifically looking at modern Western societies (Baumeister & Muraven, 1996). This is because different societies present different representations, thereby constituting different realities (Moscovici, 2001). In the process of constructing one's racialised identity, different identity aspects interact with one another within the environmental and social context (Howarth, 2002a, 2006). Thus, changes in the socio‐political context should result in changes to one's racialised identity. Importantly, social, political and historical contexts are not external factors that impact one's perception and construction of Self. Within SRA, context is a *reality* that is brought into existence through representations. SRA's theoretical framework, which draws from SIT and SRT, consists of four facets, *shared knowledge, meta‐knowledge, enacted communication* and *world making assumptions* as key tenets of social representations of the social world. Notably, one of the four facets of SRA, *shared knowledge*, will be the point of focus in this paper. Shared knowledge refers to the “qualitative epistemic transformation” (Elcheroth et al., 2011; p. 737) that understanding goes through when individual experiences changes to shared meaning, thereby becoming social fact. In sum, identification with a racialised group entails also understanding the shared meanings associated with said group.

### Racialised and National identities in context

While many psychologists do not use the two terms interchangeably, they avoid the use of the term “race” to describe a social category that is salient for most people, instead using the term “ethnicity” (Helms & Talleyrand, 1997; Howarth, 2009; Mama, 1995). Some scholars have argued that doing so leaves the persistent nature of racism unaddressed (Harrison, 1995). Resonating with Reicher and Hopkins (2001), we agree that it is advantageous to researchers if they are interested in how “ordinary people” employ concepts in the rhetorical construction of identities for themselves and others. We also respond to a critique that Billig (2014, p. 236) holds of social psychology that “general concepts become greedy concepts, devouring the individual, unique features of the social world” and the result “is less, not greater, theoretical understanding”. In the context of race research, this can mean concepts like stereotypes and attributes become greedy concepts that we use as general and universal terms, making contextually significant factors seem less important than they should be. Instead, we should use such constructs to sensitise ourselves to otherwise neglected features of the phenomena under study and to do so in such a way that recognises their socio‐cultural specificity (Hopkins, 2015). We take this to mean that a thorough understanding of the social world would require the social psychologist to abandon previously (or currently) held ideas about how the social world needs to be understood. Therefore, while we understand race as being socially constructed, situational and fluid and not a biological fact, we use the term race here without double quotes as it is reflected in participants' own discourse, and is used in a seemingly unproblematised manner in Malaysia and Singapore, mirroring the understandings of the social world of our participants.

Racialised identities are often connected to national identities (Augoustinos, 2001; Mama, 1995) yet, this interconnection is often overlooked in racial identity research. Having a national identity is to possess ways of talking about nationhood, being situated legally, physically, socially and emotionally within a homeland (Billig, 1995). Thus, one's national identity is located geographically within a country that is considered to be one's country of origin. Indeed, many national identities are also racialised identities. For example, the term “Indian” can be used to signal one's national identity and citizenship in the country India. It can also be used to describe the racialised category. However, in many multicultural societies, one's country of origin is not necessarily one's homeland, and the individual's homeland (or country where they possess a national identity) is at times a multicultural country where individuals from multiple countries of origin co‐exist. Following from the example above, although Indians form a specific racialised group in the US, the Indian diaspora could entail many different national identities.

Some important work that connects identity and social representations of different social groups demonstrates how different aspects of one's identity interact with one another within the environmental and social context (e.g. Hopkins & Blackwood, 2011; Howarth, 2002b, 2006; Khan et al., 2014). For example Andreouli and Howarth (2013), in their study of immigration in the UK, show that the repositioning of one (national) identity to another takes place within “the reified context of policy‐making and the consensual context of everyday knowledge” (p.377), connecting government policy and everyday practice. Another relevant study by Scuzzarello (2012) showed that the political opportunity structures operating in a context are important in understanding studying how the micro‐level of social interaction (by extension, racialised identity co‐construction) can be encouraged or hindered. Lukate and Foster (2023) show how changes in socio‐geographic context corresponded to, and encouraged changes to, Black and mixed‐race women's hairstyling, a performance of their racialised identities. A facet of SRA is that social representations are *world‐making assumptions* that not only constitute reality; they sometimes change reality as well. This means that social and historical contexts are not external factors that impact social representations, rather they are realities that are brought into existence throughout the social representations constructed, reproduced and mobilised. But the power of social representations extends beyond bringing context into existence, it also has, by nature of doing so, the ability to change representations which can in turn lead to changes in the institutional world (Elcheroth et al., 2011). In her research on naturalised citizenship in the UK, Andreouli (2010) exemplifies this facet by demonstrating how individuals draw on representations of Britishness to position themselves as insiders (British citizens), thereby changing their social realities as they dislocate themselves not only physically but also through the adoption of the new identity.

Importantly, Stevenson and Muldoon (2010) have shown that the socio‐political context influences minoritised and majoritised individuals differently in their construction of national identities. Therefore, it is not simply the context that influences national identity construction, rather it is important to factor in social hierarchies between racialised categories when understanding identity constructions. Indeed, research in South Africa (Durrheim & Dixon, 2010; Stevens, 1998) highlights how social hierarchies that exist between Black, white and Coloured South Africans are related to how individuals respond to changes to the socio‐political context seen in the case of desegregation. Social hierarchies complicate the relationship between race and nationality where it is easier for some people to adopt national identities if their racialised identities are part of the majoritised national identity. Yet, relatively little is known, outside of literature on South Africa, about contextualised institutional prescriptions of race on individuals and the resultant influence on the psychology of these individuals. Research that has focused on the structural influences that impact the individual's perceptions of their racialised identity has been limited, particularly within psychological research (see Andreouli et al., 2013). In addition, variability within communities, rather than between communities, has often been obscured in research on identities (Hammack, 2008). What this means for this study is that changes in one's social hierarchies (from majority to minority group) through changing one's physical location should result in changes in racialised identity constructions and levels of racialised identification.

## THE MULTIPLE CONTEXTS OF THE PRESENT STUDY

The socio‐political contexts of Malaysia, Singapore and the UK form a useful platform to understand racialised and national identity constructions. By using the examples of Malaysia and Singapore, two countries that used to be governed as one by British colonial administrators, we examine how border making has influenced the psychology of race, racialisation and racial identification. By drawing references to two non‐Western research settings, we also illustrate how expanding our research to less familiar research settings not only highlights the complexities of racialised identity construction, but also accentuates the limits of our current understanding. In sum, this paper adopts the view that the socio‐political context is a combination of its social policies (Reddy & Selvanathan, 2020) and colonial history (Reddy & Gleibs, 2019) and is also demarcated by country boundaries.

Malaysia and Singapore situate themselves as globalised countries, attracting international talent, students and investors (Rahman & Ahmed, 2014; Yeoh & Lam, 2016), laying the foundation for cross‐cultural exchange to occur and for citizens to develop international outlooks. Thousands of Malaysians and Singaporeans also migrate and take up residence in other countries yearly (Nadaraj, 2016; Yong, 2017). Until 1959, Malaysia and Singapore were ruled by the British as one entity (Malaya), and colonial legacies include a strict racial categorisation framework that is not followed in the UK. Both countries, once viewed by its people as a single entity, focused on nation building separately, creating their own communal and institutional bonds of belonging and multiculturalism (see Reddy, 2016; Reddy & Selvanathan, 2020). This process of creating distinct nations reflects a political (re)creation and reclamation of certain geographical boundaries to forge differences between the two countries (Anderson, 2020). Evenso, social policies in both Malaysia and Singapore rely on state mandated racial categorisation that ascribes a racialised identity onto all Malaysians and Singaporeans at birth, because race is understood to be biological and follows a patrilineal structure. Today, in Singapore and Malaysia, racialised identity is used to allocate resources such as education, housing and employment and is assigned by the state.

Despite these similarities, local social policies and their reliance on racial categories, and racial categorisation by the state, differ between Singapore and Malaysia. In Singapore's policy of multiracialism, the “social formula” of the CMIO model is built upon the acceptance of the four main races in Singapore—Chinese, Malay, Indian and “Other”^1^—as separate but equal in formulating most of its social policies, thus positioning Singapore as a meritocracy while different in practice (Mutalib, 2012). In contrast, a different form of multiculturalism is practiced in Malaysia where governance is defined by the political primacy of the Malays. Non‐Malays recognise Malay primacy in exchange for equal citizenship rights, at the expense of formal racial equality (Goh, 2008). Race‐based social policies in Malaysia consistently favour Malays (also categorised as *Bumiputras*, or *sons of the soil*), unlike in Singapore.

While the countries share a similar racial makeup of predominantly Malay, Indian and Chinese citizens with a number of minoritised groups such as Eurasians (dispassionately lumped together as “Others”), what is different is the numbers of individuals who have been categorised as Malay, Indian and Chinese. Malays make up about 60% of the Malaysian population, but 15% of the Singaporean population. Indians form approximately 7% of the population in Malaysia and in Singapore. Chinese are a minority in Malaysia where they make up about 25% of the population, but they are a majority in Singapore with about 75% of the population. Malays and Chinese differ in minority and majority status in Malaysia and Singapore, while Indians are a minority in both countries. Power relations and social hierarchies between groups also influence boundaries between the races and this impacts the construction and maintenance of racialised identities in each country. Majoritised‐minoritised positions are therefore important in understanding intergroup relations and identity constructions in the two countries. Political power lies with majoritised communities within both countries, but majoritised communities in each country (such as the Chinese in Singapore) do not necessarily have power in the regional (Southeast Asian) and global contexts (Soon, 1974). Ultimately what this shows is that being Malay, for example, in Malaysia, would afford very different outcomes to being Malay in Singapore because of differences in political ideologies, and its subsequent social hierarchies.

The UK is one of the countries that sees many Malaysians and Singaporeans taking up temporary or permanent residence (Office of National Statistics, 2013). However, race is constructed very differently by the UK state. Racial (or rather, ethnic^2^) categorisation in the UK results from self‐identification when filling out a government form. Residents have the option of choosing an ethnic label for themselves within institutional systems (when filing up an intake form at the general practitioner's office for example), and having a system of race‐based social policies would be considered illegal racism. Yet, racism exists within the UK context precisely because of the co‐constitution of meanings about identities and their ideological hierarchisation and policies discriminate even if they are not race‐based (Andreouli & Howarth, 2013). Thus the UK presents an interesting research context to study how Malaysians and Singaporeans construct race when their social worlds change, as they would not need to use racialised categories imposed by the Singaporean and Malaysian government to access resources in the UK. Furthermore, they have the option of giving themselves a racialised identity that they self‐identify with.

## PRESENT STUDY

### Method

Given the limited scope of research on identity content as described earlier, a qualitative focus group study (*N* = 10) was conducted to understand how, and why, racialised identity content changed across socio‐political contexts.

### Materials

We developed a semi‐structured focus group topic guide (Table S1) that was used in all three locations (Singapore, Malaysia and UK). The interviewer asked nine open‐ended questions, which were often followed up with explanatory probes. For example, the probe “how representative of your ethnic group are you?” followed the question “how does your idea of your ethnicity/race differ from others in your ethnic/racial group”.

### Epistemology & ontology

The authors acknowledged the presence of multiple, constructed realities, so as to work within the limits of “reality” as viewed by participants. For example, the interviewer took direction from the participants in the usage of ethnicity or race. That is, if the participant used the term race, the interviewer would also use the term race. Of course, the irony of using the same categories that political institutions use, in our study, is not lost on us. However, following Radhakrishnan's (1996) view on working within the treacherous binds and Hall's (1996) position that until such a time where such concepts are no longer useful in understanding the people we study, the social scientist needs to engage with them, we have used maintained the use of the same categories, albeit in a critical manner. We acknowledge the inherently problematic view of clearly demarcated racialised categories, and as a result, what is seen as distinct, separate and exclusive racialised identities. Nonetheless, we use the categories Indian, Chinese and Malay as participants understand and use it. This is in line with our critical realist ontological and pragmatic epistemological framework (Willig, 1999), which is observing reality, as participants perceive it to be. We adopted the view that meaning is participative and not simply produced by the participants, but that the contribution of the researcher to the construction of data corpus needs to be acknowledged.

### Sample

The sample consisted of a total of 39 participants. There were 16 Malaysians and 23 Singaporeans in total. Four focus groups were conducted in Singapore, two focus groups were conducted in Malaysia and four focus were conducted in the UK. Each focus group was conducted with different participants. All focus groups discussed how changes to their socio‐political context, such as moving from Singapore to the UK, would influence racialised identities. Participants are identified by their pseudonyms, nationality and racialised identification, where they wished to be identified as such.

### Analytic approach

The data was analysed using thematic analysis (Braun & Clarke, 2006) at the semantic level, where codes and themes were identified within the surface meanings of the data (Patton, 1990). The data was managed using NVivo. As we were examining how, and why, racialised identity constructions differed between Malaysia, Singapore and outside of these socio‐political contexts, we identified data that addressed this question. We then coded the data both deductively (guided by theories and empirical research discussed) and inductively (we identified issues that were brought up by participants themselves). An example of a deductive code is “Comparison with home country”, and an example of an inductive code is “Leaving the country to be recognised as a citizen of the country”. Prior knowledge of theoretical frameworks gave us the analytical edge in contextualising the analysis of data.

### Analysis

Three overarching themes (Table S2) were identified in analysing why racialised identity constructions changed when the socio‐political context changed; “*Stigma and Stereotypes influence construction*”, “*Racialised identification does not always transcend country boundaries*” and “*Cultural reference but not identification*”. These three themes illustrate how participants' *experiences* of racialised identities in different countries led to the *re‐construction* of the meaning of their racialised identities. The extracts below explore these themes in detail.

### Stigma and stereotypes influence construction

Participants living outside of their countries of origin discussed how they could change the construction of their racialised identities in different socio‐political contexts, especially one that was different to where they first identified with the racialised category. In this paper, we refer to this as the “home country”. This was particularly significant with regard to the Malay racialised identity. For example, one participant mentioned that she could define what it meant to be Malay, when she was in the UK, compared to when she was in Singapore. When she was growing up in Singapore, she had to align her Malay identity with reference to dominant representations.

#### Extract 1

Zara I find it easier to be Malay here. [Sofia agrees] I love being Malay here. I can actually feel like I can actually be stuck here. (…) But in London, you see all these buildings, look how old they are, and there's an appreciation of culture, revisiting stuff, and recreating stuff, like being inspired from your past, whereas Singapore is just like, knock down, knock down.

First author Why is it easier to be Malay here?

Zara Um, also, you're not stereotyped.

Zara, Singaporean Malay.

Zara points to the lack of stereotypical constructions of Malay identity in the UK allowing her to change the contents of the Malay identity. She can construct her Malay identity alongside what she perceives as important in the new context, that of nostalgia and recognition of the worth of one's culture. Sofia, another Singaporean focus group participant shares her view on being able to identify with the Malay identity more easily outside of Singapore because of the ability to change the content of the identity.

In another focus group conducted in Singapore, participants discussed some of the stereotypical constructions of the Malay identity.

#### Extract 2

Zainal Being Malay, I think, Malays have a lot of negative stereotypes, for sure.

Shan Yah.

Zainal It's very very bad. It reinforce(s), especially If you're not sure of who you are, it reinforces your mentality. Like if you're a Technical student, 90% of them are Malay, you are an SCDF,^3^ confirm Malay, Police Force, also Malay, Navy is no Malay.

First Author How does that make you feel?

Zainal Man, I feel kind of upset, honestly speaking.

Mika Yah, Yah.

Zainal, Singaporean Malay, Shan, Singaporean Indian, Mika, Singaporean Indian.

Participants, regardless of whether they self‐identified with the Malay identity, knew of the constructions of the Malay identity as being limited to specific jobs in the armed forces and found mainly in technical education streams in Singapore. This not only upsets Zainal, a Malay‐identifying Singaporean, but also Mika, an Indian‐identifying Singaporean.

A difference in negotiating their Malay identities is thus visible when we compare these two extracts that take place across country boundaries. Zainal feels constricted by the representations of being Malay in Singapore. Being in UK that does not reinforce these representations is seen as liberating for Zara and Sophia. Their view is elaborated in another focus group discussion conducted in London.

#### Extract 3

(…) the Malay community being here, they like being here, they are open to new experiences, the pressure to confirm is not as high as in Malaysia, where there are Malays everywhere. But here, there is also a group which I see them for Raya, they still wear Baju Melayu, so you can still practice your Malay culture, at the same time, you can do your own stuff, you're free to do it, it's quite anonymous. Ilan, Malaysian Malay‐Chinese.

The anonymity associated with being outside of the country of origin^4^ encourages individuals to alter their constructions of Malay identity according to what suits them. In changing contexts, they are free from social expectations of how the identity needs to be enacted and communicated. The “new” constructions of their racialised identities in the UK is compared to the negative stereotypes of Malays in Singapore and the societal pressures of enacting Malay identity in Malaysia. Contrary to other research on migrant minority identities as devalued and stigmatising (see Chryssochoou, 2004), here we see that migrating to a new country offers participants a more positive view on their racialised identities. The new country of residence presents less restrictive options for these participants. This is not to say that the participants did not experience racism in the UK.^5^ Rather this point of reflection occurs because participants are comparing their experiences of being limited in their expression of their Malay identities in Malaysia and Singapore. However, freedom from stigma and stereotypes is only part of the reason why participants changed the construction of their racialised identities.

### Racialised identification does not always transcend country boundaries

The change in racialised identity construction is in part due to the perspective that individuals who share the same racialised heritage do not necessarily belong to the same ingroup.

#### Extract 4

There is a stark difference between those who are Singaporean Indian, and those who are non‐Singaporean Indian. I think if you look at it as ingroup‐outgroup boundaries, the ingroup is more, not really your ethnicity,^6^ but more of Singaporean. Bala, Singaporean Indian.

Bala redraws the boundaries of the ingroup from one that is limited to his racialised identity, to another that is hyphenated with his national identity. We see that his self‐racialised identification is embedded in his national identification. The distinction was also extended to the Chinese identity.

#### Extract 5

Trina Or like even Singaporeans are totally different.

First Author OK, so how different?

Trina Just different.

Louisa Yah they are just different.

Trina They speak the same way we speak but different

Amit The culture is different.

Trina the mentality. (Group laughs)

Trina, Malaysian Chinese, Louisa, Malaysian Chinese, Amit, Malaysian Lain‐Lain.

7

Here, participants in another focus group conducted in London discuss how Malaysian Chinese and Singaporean Chinese are different. Not only did participants distinguish between racialised identity members in Malaysia and Singapore, they would compare their racialised identities against individuals from the countries where the racialised identities originated.

#### Extract 6

But about the time when I started realizing my more Indian Singaporean self, was when I was in secondary school, and most of my Indian classmates were India‐born. And that was when, when you talk to them, and suddenly, you realise there's something called a caste system, and the way that you speak Tamil to them is different, they all eat vegetarian food and home, and I realized how different I am for them, and my experience of growing up is very different from theirs. Revathi, Singaporean Indian.

Revathi's perspective of the Indian identity is different from how Indians from India construct their racialised identities, leading her to argue that she is different from others from her country of origin. Indians and Chinese in Malaysia and Singapore have their roots in India and China respectively. Yet, participants did recognised individuals who are first generation Malaysians and Singaporeans of Chinese and Indian descent as being the same as Chinese and Indians who are currently living in those countries. Another participant in a focus group conducted in Malaysia shared this sentiment. Here, Sarojini echoes a similar sentiment to Revathi.

#### Extract 7

No, I think there is a difference between what you identify with, and also the country of origin right? Like, of course, we have, yeah, my mother is Chinese but she was born here in Malaysia. Yeah my maternal grandparents are actually from mainland China, but you know, I don't feel any affinity with China as a country, and neither do I feel any affinity, you know, to India as a country. Sarojini, Malaysian Chinese‐Indian.

Sarojini, a multiracial individual describes a lack of closeness to the countries where her grandparents have come from, and by extension, her racial identity roots. Again here we see how she grounds her racialised identification, and that of her mother's, within her national identity. For some participants these differences within the same racialised identity came as a surprise.

#### Extract 8

So, that got me a bit more interested in Chinese culture, and I started to travel to Chinese places for vacations. But when I travelled out, I realized that the Chinese culture that I expected there is actually quite different from what I expected there, and I found myself drawing a line between I'm Chinese and I'm Singaporean Chinese, because we're so different in our habits and even our mindsets, and yeah, so. I'm quite glad that I'm a Singaporean Chinese and not a Chinese Chinese. Ray, 27 Singaporean Chinese.

Ray, on the other hand, travelled to the country of origin (China, in her case) only to realise that what she assumed was a common construction of Chinese identity was instead not enough to share a common group identity.

What these four extracts show is a desire for participants to distinguish themselves from the racialised identities of countries where their ancestors originated. Their constructions of their own racialised identities are embedded within the national identities, and participants find ways to contrast their identities by highlighting differences in thinking and lived experiences. While the countries of origin remain the same (i.e. Indian) what it means to be Indian is different in India, Malaysia and Singapore. However, Ray's perspective also tells us something about the similarities between people that share the same racialised identity.

### Cultural reference but not identification

While Singaporean and Malaysian participants did not identify with people from China for example, they found themselves connecting with Others who shared their racialised identity when they were in the UK.

#### Extract 9

Jing Wei I think Chinese Malaysians are more, they seems to me like they can make friends with other Chinese from other parts of the world.

Aadil I agree.

Jing Wei Like maybe Taiwanese, they can easily like, talk to them, they can form group very easily, like people from Hong Kong, Singapore, they can form group very easily, from what I can see.

Jing Wei, Malaysian, Aadil, Malaysian Malay.

Jing Wei speaks of Malaysians who identified as Chinese showing an inclination to connect with Chinese from other parts of the world. There exists a platform in the UK to focus on the commonality of Chinese heritage. Yet, even within this, we see that Jing Wei distinguishes them by nationality (Taiwan, Hong Kong, Singapore) rather than viewing them as individuals who have ancestors from the same country of origin. What is interesting also is that Jing Wei does not name people from China specifically, but does name Chinese from countries other than China (Taiwan, Hong Kong, Singapore). His connection is limited to people from these countries and not from China, like Ray^8^ in Extract 7, perhaps because he perceives that Chinese from these other countries form the diaspora, and the diaspora shares similar constructions of Chinese identities compared to the country of origin. Here, we see a clearer relationship between identification and content of identity. Racialised identification, for these participants only extends to individuals who share the same national identification because they share the same identity content.

## DISCUSSION

The present paper sets out to explore the social‐psychological relevance of context for how geopolitical relations and histories become embedded within identities. In particular, we focused on examining how, and why, racialised identity constructions, among Malaysians and Singaporeans changed as they moved, and existed, across different socio‐political contexts. What is clear from the analysis is that participants have a nuanced understanding of the multiple countries that their racialised identities originate from, are constructed in and are managed in. Each country marks a different socio‐political context that has different social hierarchies and social policies, to name a few of these differences. These contexts in turn intersect with, and impact, how race, ethnicity and nationality becomes embodied, and how they are more or less flexible. It is this embodiment of the various aspects of context that we refer to as “*politicized psycho‐geographies*” detailed below.

Previous research has shown that identity construction is influenced by the everyday engagement with social policies in a socio‐political context (Reddy, 2017), and this paper extends this by defining the context for the racialised identity, distinguished here as country of origin, country of birth/citizenship and country where identity is negotiated. This definition has merit in deepening our understanding of the changes in the psychology of racialised identity construction. To further this point, we see a distinction in the ways racialised identities are constructed in the different types of countries. The analysis shows how racialised identification differs between “home” countries (country of birth/citizenship) that participants were brought up in, and countries outside of these “home” countries. Extract 1, Extract 3 provide us with some insight as to why participants change, and importantly why they perceive that they have the ability to change, the constructions of their racialised identities. Freedom from stigma and stereotypes associated with dominant representations in the home countries drove some of the change in construction of their racialised identities as participants felt stifled by the stereotypical constructions of their racialised identities in Malaysia (country of origin) and Singapore (“home” countries). This is of course complicated by Malaysia being a country of “origin” and “home” for Malaysian Malays, who share the freedom of re‐constructing their Malay identity in the UK with Singaporeans. These stereotypes are rooted in colonial constructions of the three main racialised identities in the two countries and continue to endure in the psychological imagination of the Self and Other in these two countries (Reddy & Gleibs, 2019).

Another reason for the change in individuals' construction of their racialised identities could be because they see racialised identities as being hyphenated with their national identities. Individuals who identified as one racialised identity in their “home” countries (Chinese for example) did not necessarily identify with other Chinese individuals outside of their home countries (in the UK for example). This is because, the core reason for their identification is the socio‐political context (Chinese Singaporean, rather than only Chinese) given that the context requires individuals to identify racially. The context (Singapore) enforces a racialised identification for all citizens and it is strategic for Chinese in Singapore to identify as such given the political power that Chinese hold within the country (Reddy, 2017). When the context changes (UK), and individuals no longer need to strategically identify as Chinese, the racialised identity is not strong, and individuals find value in distinguishing themselves as Singaporean Chinese, rather than Chinese alone. Here we see the preference to be identified as a dual identity (Hopkins, 2011) with both national and racialised identities combined. Extract 4, Extract 7 show that participants distinguish themselves from individuals who share the same country of origin, and prefer to identify themselves as *both* racially and nationally. This parallels Tajfel's (1978) early claim that the extent to which individuals see themselves and Others in terms of group membership, and the extent to which they personally identify with the social group to which they belong, individuals tend to act towards Others as group members rather than unique individuals. What this means is that participants would consider individuals they meet in other countries who share *both* the same racialised and national background as themselves, as members of the same ingroup. This is elaborated further in Extracts 8 and 9, which shows how participants draw on similar cultural references from their racialised identities to connect with Others who share the same racialised identities, but therein lies the extent of “sameness”. Participants still fall back on the hyphenated nationality‐racialised identity to distinguish between members of the same racialised group. This highlights that participants do not represent racialised groups as homogenous, and racialised identities are often embedded in national identities.

One's preference to identify with a racialised identity is influenced by common understandings and constructions of the said identity, as shown in SIT research (Haslam et al., 1999). Yet here, we see that the strength of one's identification with a said identity has a part to play in their desire to change the identity contents. Rather than choosing another facet of their self‐concept to identify with, as understood by the SIT concept of *social creativity* (Tajfel & Turner, 1979), where the individual compares the ingroup and outgroup on another dimension such as gender instead of race, through the analysis we see a commitment on the part of the individual to change the devalued contents of their identity because they identify strongly with the identity category. Therefore, we see a connection between identification and identity content beyond that outlined by Howarth (2002b) by factoring in strength of identification.

## CONTRIBUTIONS

The present paper makes three significant contributions to the study of racialised identities. Firstly, we put forward a theoretical suggestion through these findings. Echoing Howarth (2002a), (2002b), identification and content need to be understood hand in hand and here we see that identity strategies that the individual engages in can be elucidated when exploring these two social psychological concepts relevant to racialised identities, *together*. We suggest that identification is indeed still relevant, and in fact bears some relation to the content of the identity. Indeed, the individual who wishes to identify with a group asks the question “who am I?” in terms of characteristics that they think they share with other group members (Reicher et al., 2010). In other words, the individual taps on the shared knowledge of what the identity means to decide how much they want to identify with that identity. Thus a key component of identity construction is identifying with the said identity as has been discussed elsewhere (Howarth, 2002a). What we suggest here is that this relationship between the strength of identification and identity content should be embedded within the SRA concept of shared knowledge. Social representations are intertwined with identity construction (Jovchelovitch, 1996). Jovchelovitch clarifies this to mean that “there is no possibility of identity without the work of representation, just as there is no work of representation without an identificatory boundary between the me and the not‐me” (p. 126). To add to this position, we offer an extended conceptualisation of shared knowledge to include not only what is shared by those who identify with the identity in question but also what becomes of the shared knowledge in different settings depends on how much one identifies with it, highlighting the critical potential of shared knowledge within the SRA framework.

Secondly, the paper directs focus of psychological studies of racialised identities onto the content of identities rather than the magnified, though informative, focus on the strength of identification of racialised identities. It shows while racialised identity categories (as political categories) remain the same across different contexts and are enduring concepts, the content of these categories differ; thus participants have different understandings of these racialised identity categories as they exist in different socio‐political contexts. This paper highlights that one's racialised identity may not change across contexts, but what individuals construct that identity to be changes across (symbolic representations of) context. There is an assumption that what the individual associates with a racialised identity is constant across space. We know that identity is fluid, and individuals change what the identity means for them in different situations. Yet, what this paper demonstrates is that identities can change across space. For example, what is perceived to be Chinese, by a Chinese‐identifying individual, is meant to be constant regardless of context. However, this paper asserts that the socio‐political contexts (both symbolic representations of and experiences of changes) influence individuals to construct their identities differently, thus changing what it means to be Chinese in different contexts. Unlike Perkins (2006) research, which shows the differences in the meaning of the same identity category among a diverse group of people who all identify with the said racialised identity in the same socio‐political context, here we see that a change in the socio‐political context triggers a change in the meaning of the racialised identity.

This leads to our point on the conceptualisation of the socio‐political context, which is our third contribution. Our initial definition of the socio‐political context was limited to political (multicultural) ideologies of governments that were also differentiated by country boundaries. From the findings of this paper, we see that individuals construct their racialised identities distinctly in different countries. This distinction is made between country of origin, country of birth/citizenship and country of residence where identity is negotiated. Identities are constructed and re‐constructed through debates and social practices, and it has been said that this contested process is not limited by cultural or geographical boundaries (Hopkins & Reicher, 2011). In fact, representations of the socio‐political context are shaped by practices, such as discriminatory acts and stereotyping (Andreouli, 2010; Howarth, 2006). Herein we see that while cultural boundaries remain the same insofar as participants find common cultural references, but newer boundaries are created within the same racialised identity because racialised identities are constructed with national identities that have been cleaved by the colonial imagination. Social psychology has been criticised for not addressing the “active nationalization of the Self” (Hopkins & Moore, 2001; p.240), and this paper responds to these critiques by outlining how national identities are mobilised in the construction of racialised identities. We argue that by hyphenating racialised identities with national identities in the everyday construction of racialised identities, individuals politicise their racialised identities further by creating more boundaries that are not just geographical, but also relational. The countries (Malaysia, Singapore, UK, China and India) are delineated as country boundaries, but for the participants there is an additional psychological imagination of the differences between the countries as differentiated by origin, birth, citizenship and residence that is influenced by colonisation and migration. That is, how each country relates to their personal location vis‐à‐vis their racialised identities. Furthermore, social hierarchies change with respect to one's racialised identity in each of these countries.

Thus, the paper evidences and gives us a language for how social psychology of context and in context relates to geopolitics because it shows how a shift in countries (and their associated political systems) create hierarchies of belonging around racialised identities that are related to the geopolitical histories and relationships between nations. We offer a social psychological conceptualisation of context that articulates that context is a psychological interpretation of oneself in relation to different political systems, social hierarchies, and colonial histories as embedded within a country boundary. To this end, the content of a racialised identity changes across country boundaries because the context forces one to (re)interpret what their identity means for themselves. What this means for future psychological studies is that racialised identities cannot be used in homogenous terms across country boundaries.

*Politicized psycho‐geographies* are aspects of the socio‐political context that include both the realms of the political elite as well as the everyday politics, and can be mapped onto different geographical locations. It is ultimately the psychological relationship between individual, politics and country. The field of political geography focuses on “the geographical and spatial dimensions of politics and the political” (Political Geography, 2024) and the social psychological study of race in the socio‐political context would no doubt benefit from a closer connection with this extensive work (see also Koch & Passi, 2016). *Politicized psycho‐geographies* goes beyond this focus because it takes into account the active construction and co‐construction of racialised identities within these different geopolitical, historical and socio‐political contexts—thus, taking a social psychological perspective. Racialised identity construction is inherently relational and rooted within a conceptualisation of culture that includes the colonial institution (Reddy & Gleibs, 2019). In sum, we underscore the importance of everyday racial politics, colonial history as well as the more active notion of politicisation of the psyche, in our conceptualisation of *geographies* as both *politicized and* deeply *psychological*.

## CONCLUSIONS

Racialised identities are often discussed from a singular, monolithic perspective, and compared across contexts in a similar fashion in psychology. For example, cross‐cultural research often compares how individuals acculturate or assimilate when they move to a new country. Chinese migrants in America are often studied as a homogenous group, and details regarding the countries they have migrated from are often left out (e.g Lieber et al., 2001; Schnittker, 2002). The present study shows that the diaspora might have different conceptions of these identities compared to those living in their country of origin at the everyday level. What it means to be Chinese to a Singaporean, is different to a Malaysian's construction of Chinese identity. Because Chineseness to a Singaporean is embedded in the Singaporean context, sharing Chinese heritage is not enough to forge a common ingroup identity in a different country. Racial categories are entrenched within the countries that they originate, that they are formed in and that they are negotiated in, and each of these countries present different constructions of the same racial identity. Therefore, race is not merely a category within a national context. It is a system of representations that “set(s) out the field of activity and inform(s) the members of social systems of their rights & duties; of a sense of belonging” (Moscovici, 2001; p.21). It binds people together, within the context that it was formed in. This paper shows that socio‐political context is not just a geographical or spatial concept, but it is a state of being that anchors one's world view and one that they carry over when they cross spatial contexts. Indeed, Jovchelovitch (2007; p.48) outlines how the individual themself is a “multidimensional context” comprised of both the body and the mind that is “socially, culturally, and historically located”. This is a significant finding and bears relevance to work done within social psychology given the increasing research focus on migration taking place in the globalised world today. Care needs to be taken especially when racial categories are manipulated as variables (Helms et al., 2005). This nuanced perspective of the socio‐political context and migrant populations will be useful in understanding issues of cohesion, acculturation, intergroup relations and above all, racialisation, in the increasingly diverse multicultural societies we live in.

## AUTHOR CONTRIBUTIONS

**Geetha Reddy:** Conceptualization; methodology; writing – review and editing; writing – original draft; project administration; formal analysis; funding acquisition; data curation. **Ilka H. Gleibs:** Supervision; conceptualization; writing – review and editing.

## CONFLICT OF INTEREST STATEMENT

There are no conflicts of interests.

## ETHICS STATEMENT

This study was carried out in accordance with the recommendations of the British Psychological Society (BPS) ethics guidelines and approved by the Chair of the Department of Psychological and Behavioural Sciences ethics committee, as well as Research Degrees subcommittee of the London School of Economics and Political Science, United Kingdom. All participants gave written informed consent. Confidentiality was emphasised and participants details were anonymised accordingly. To protect participant confidentiality, all interview participants were given pseudonyms during the transcription process.

## Supporting information


Data S1.

## Data Availability

Data available on request due to privacy/ethical restrictions.

## Footnotes

^1^
The category of “Other” encompasses all who did not fit into the categories Chinese, Malay or Indian, and includes all European heritages and nationalities as minority groups (Hill & Lian, 1995).

^2^
Here we refer to ethnicity, as this is the term used in UK public understanding and governmental discourse.

^3^
SCDF stands for Singapore Civil Defence Force, a government organisation that primarily provides emergency services such as firefighting, and emergency medical services.

^4^
Malaysia is the country of origin for the Malay identity.

^5^
We would go so far as to say that the focus group discussions created a particular context such that discussions were limited to understanding tensions and ease associated with their Malaysian and Singaporean racial identity constructions. Broader discussions of racism in the UK were limited.

^6^
Participants used race and ethnicity interchangeably and extracts here reflect participants' speech.

^7^
Lain‐Lain is a category given to individuals who do not fit clearly into Bumiputra, Chinese and Indian category. In this case, Amit identifies both as Lain‐Lain and Punjabi.

^8^
Ray and Jing Wei were participants in different focus groups carried out in London.
