# Supplementary material for: Context as politicised psycho‐geographies: The psychological relationship between individual, politics, and country
Source: Br J Soc Psychol. 2025 Jan 28;64(2):e12848. doi: 10.1111/bjso.12848 (PMC11773681; doi:10.1111/bjso.12848)
Supplement: Supplementary file 1 — Data S1. [file BJSO-64-0-s001.docx]

# Table S1

## Interview Schedule

| Main Questions | Prompts |
| --- | --- |
| Thank you for agreeing to take part in this research study. (Author introduction) I hope to get as much information as possible so please share as many details and opinions. There are no right or wrong answers. I am interested in your opinions. The discussion will be video recorded so that it can be transcribed later. This will help me understand better what everyone is saying. No names or personal identifiers will be used at any stage of the analysis. All information will be kept confidential and will be used for research purposes only. Are there any questions at this stage? Ok, before we begin, could you share with me why you agreed to take part in this interview? |  |
| Ok let’s go around the group and introduce ourselves. We can state our names (or names that you would like to be called in this group), pronouns that you would use to call yourself, and the item that you have brought along that encapsulates your ethnic identity. |  |
| What racial category was used to describe you when you were born? |  |
| What are the ways you explored your Malay/Indian/Chinese Identity? | What makes you Malay/Indian/Chinese? Language? Food? What does the Malay/Indian/Chinese culture mean to you? |
| How does your idea of your ethnicity/race differ from others in your ethnic/racial group? | How representative of your ethnic group are you? Do you find it easy to be a member of your ethnic group? |
| When you see another person of your ethnicity/race, what language do you speak to them in? | How do you decide if someone you have never met is of your ethnicity/race? |
| What do you think of interethnic marriage? | Do you have friends/other family members (not from your immediate family) who are mixed? |
| Suitable Vignette from list | What do you think about this statement? |
| Malaysia/Singapore is seen as a multiracial country. What makes it multi racial? | What are some ways that Malaysia/Singapore is multiracial? What is the importance of Malaysia being multi racial? |
| How similar is being Malay/Indian/Chinese in Malaysia/Singapore and London? |  |
| We are now at the end of our discussion and I would like to get some feedback from you. Considering all the issues discussed this afternoon, which do you feel are the most important issues discussed? | Have we missed out any important issue? |

# Table S2

## Thematic Analysis

| **Code** | **Basic Theme** | **Description** | **Main theme** |
| --- | --- | --- | --- |
| Malaysia vs Singapore | **Differences between Malaysia and Singapore** | Race is understood and experienced differently in Malaysia and Singapore | **Racialised identification does not always transcend country boundaries** |
| Leaving the country to be recognised as a citizen of the country | **Change of socio-political context means being able to identify with national identity** | Participants discuss being able to identify with nationality outside of country of citizenship/country of birth |  |
| Local vs London | **Differences between home country and London** | Racialised identities are experienced and managed differently between home country and London |  |
| Comparison with “home” country |  |  |  |
| “Outside vs inside” |  |  |  |
| Superordinate identity | **Identification beyond race** | Other identities such as national identity are preferred |  |
| Freedom to create racialised identity outside of local context | **Freedom to re-construct racialised identity outside of “local” socio-political context** | Participants talk about being able to construct their racialised identities more freely in London compared to Malaysia or Singapore |  |
| What does it mean to be Malay | **Change in identity constructions across politicised psycho-geographies** | Participants have different constructions of racialised identity differentiated by country of origin, country of citizenship/birth and country of residence |  |
| What does it mean to be Chinese |  |  |  |
| What does it mean to be Indian |  |  |  |

| Diversity in Indian categorisation | **Indian identity more complex than Category** | Identity is complex, category is simplified | **Racialised categories used by government is limiting and essentialising** |
| --- | --- | --- | --- |
| Not fitting into boxes | **Not fitting into boxes** | Participants do not fit neatly into racial categorisation framework |  |
| Chinese Chinese vs Western Chinese | **Formation of different boundaries within same racialised identity** | Racialised identity is not homogenous among members of same racialised group |  |
| Formation of group boundaries |  |  |  |
| Race is important in Malaysia | **Importance of race in both Malaysia & Singapore** | Race is pervasive in both countries and participants need to engage with racialised identity frameworks in their daily lives |  |
| Race is important in Singapore |  |  |  |
| Distancing self from existing stereotypes | **Distancing self from existing stereotypes** | Racial Stereotypes lead participants to distance themselves from racialised identity | **Stigma and Stereotypes influence change in racialised identity construction** |
| Stereotypes, prejudice, discrimination | **Stereotypes, prejudice, discrimination** | What are the different racialised stereotypes? |  |
| Common cultural references as a group boundary | **Cultural references unite members of same racialised identity** | Participants draw from similar cultural experiences to connect with members of the same racialised identity across country boundaries | **Cultural Reference but not identification** |
| Sharing same race is a bridge for social interactions | **Racialised identity connects people from diaspora** | Sharing the same racialised identity can mean connecting with people in different countries |  |
| Chineseness/Indianess/Malayness connects people across national boundaries |  |  |  |

## Thematic map
